# Ageing in Saudi Arabia: new dimensions and intervention strategies

**DOI:** 10.1038/s41598-022-25639-8

**Published:** 2023-03-10

**Authors:** Asharaf Abdul Salam

**Affiliations:** King Saud University Center for Population Studies, Riyadh, Saudi Arabia

**Keywords:** Developmental biology, Health care

## Abstract

Ageing process of population passing through demographic dividend in many of the Arab countries, including Saudi Arabia, where the demographic transition process entered a progressive stage. This process has been accelerated with rapid reductions in fertility caused by various changes in the socio-economic and life style dimensions. Researches on population ageing in the country are rare and thus this analytic research aims at exploring population ageing trends at the backdrop of demographic transition to help build up demanded strategies and policies. This analysis explains a rapid native population ageing especially on absolute size: an increase in line with theoretical demographic transition process. Consequently, structural changes in age distribution accompanied a change in age pyramid from an expansive shape of the late 1990s to a constrictive shape in 2010 and further shrinking by 2016. Obviously, various age related indices—age dependency, index of ageing, and median age—exemplify this trend. Still, the old aged population remain static in terms of percentages or indices, exemplifying that the movement of age cohorts continue in the early ages shall reach old age, soon, in this decade: hence, characterizes retirement boom and multiple pathologies compressed to last years of life. Thus, this is an ideal time to prepare for challenges of ageing, learning from the experiences of nations confronted with similar demographic trends. Old aged population deserves care, concern and compassion to ‘add life to years’ with dignity and independence. Informal care mechanisms, especially families, play a vital role on this behalf, and so, deserve to be strengthened and empowered through welfare measures, rather than turning to improving formal care system.

## Introduction

Saudi Arabia, the largest country in the Arabian Peninsula, has gone through several demographic changes especially in fertility and thereby age distribution and ageing scenario pressurizing other sectors such as education, health, and women employment^1–11^. The overwhelmingly young population succeeded by a hike in median age accompanied by declines in death rate, both overall and infant made gains in life expectancy^5,12^. Several changes complemented the demographics of fertility with geographic and parity differentials determined by education and age at marriage. All these developments influenced ageing process and life style of old aged persons including living arrangements, health, family support mechanisms, and satisfaction^4,8,9,13–20^.

Increases in age at marriage and widened age gap between husband and wife along social, educational, and labor force transformations impacted marital life leading to pronounced widowhood, acute morbidity in old age and thus loneliness demanding formal care mechanisms: not only medical care but also wellbeing and quality of life^13,21–23^. Thus, the whole dynamics including religious practices, community variables, marriage pattern, child bearing preferences, and therefore the existing demographic lag are under debate in the context of population ageing to create age care policies and programs^18,19,24^.

The ongoing structural changes in population of Saudi Arabia, limited to natives, have many positive sides such as gaining life expectancy; improving demographic dividend (a large proportion of working age with a smaller proportion of dependents, considered to be a window of economic opportunity, as stated^25,26^; improving health; strengthened health care system; and improving education and labor force participation: most of them are similar to countries around the globe^3,14,27^. All these positive aspects of population lead to another paradigm change, a social challenge—population ageing^22,28^. Probably, it leads to compression of morbidity at the last years of life and ageing of the aged population, as the dividend moving to upper levels of population structure^2,4,29^.

At this juncture, age care mechanisms appear as important in replacing maternal and child care even adolescent and reproductive health care, in the coming decades. These issues might lead to erosion of religious, social and familial values of caring older persons along weakening of informal care and filial care responsibilities^26,28^. This gives importance to emergence of formal care mechanisms, under conditions of living arrangements characterized by sub-nuclear families in place of existing traditional community living impacting need fulfilment and satisfaction in retired old aged life, as pointed out^14,30–32^. Apart from that, there are older persons without kith and kin to depend upon and also sexagenarians caring for octogenarians and centenarians. In this context, formal support systems including welfare measures are essential to protecting needy older persons, where primary health care centers play crucial roles^3,26,33,34^.

Here, in this research, an overview of the country ageing situation is made. Beyond a situational assessment, this research brings out suggestions to confront this development, futuristically. It is hoped that these assessments and suggestions would help to streamline policies and programs to meet the challenges of ageing and age care in the country.

## Data and methods

This research makes use of data from six sources published by General Authority for Statistics, Saudi Arabia, namely census (1974, 1992, 2004, 2010), demographic surveys 2016, Elderly survey 2017, and household health survey 2018. While age distribution was extracted from the first two sources morbidity, mortality and support requirements were extracted from the other two sources. The fifth source of data was of age distribution estimates for 2020 by the General Authority for Statistics. In addition, online data from https://www.macrotrends.net/countries/sau/saudi-arabia accessed to plot demographic transition (sixth source). Data analysis, in MS Excel, followed as below

- Percent distribution (total population in the denominator so that the total of males and females equals one hundred) for plotting age pyramid.
- Percent distribution of population by broad age groups.
- Age dependency ratio as 65 + years to 100 persons of age 15–64 years.
- Median age of population as ${\text{Median}} = {\text{lmd}} + \left( {\frac{{\frac{{\text{n}}}{2} - \Sigma {\text{fx}}}}{{{\text{fmd}}}}} \right) \times {\text{i}}$
- Index of ageing as 65 years and above to 100 persons of age less than 15 years.
- Young-old to old-old as 65–74 years to 100 persons of age 75 years and above.
- Median age of persons aged 65 years and above as ${\text{Median}} = {\text{lmd}} + \left( {\frac{{\frac{{\text{n}}}{2} - \Sigma {\text{fx}}}}{{{\text{fmd}}}}} \right) \times {\text{i}}$
- Compression morbidity as number of persons with a morbid condition in an age group to 100 persons of the same age group.
- Older persons needing support as number of persons needing any type of support in an age group to 100 persons of the same age group.
- Percent of deaths of 65 years and above as number of deaths in the age group to 100 persons in the same age group.

### Ethical approval

As this research is not based on primary data collected from any human beings, animals or other living organisms, ethical approval is not applicable. This research is based on classified data published online**.**

## Results and discussion

Results of analyses and its discussions are organized into various subsections: the last one explains the proposed programmatic changes.

## Ageing in absolute numbers

**Figure 1:**
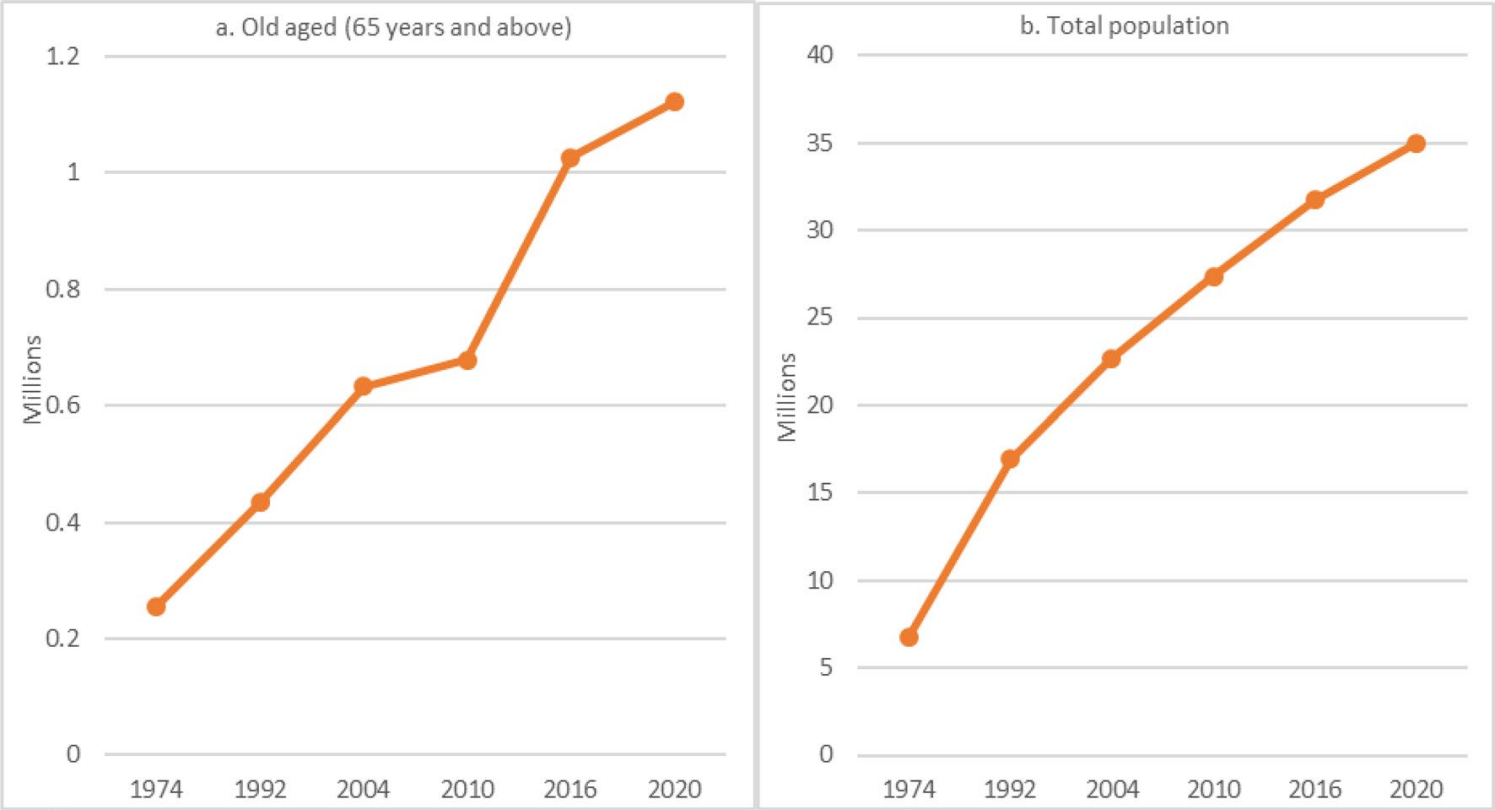
Growth of old aged versus total population.

Population of Saudi Arabia grew from a mere 6,167,308 in 1974 to 27,136,977 in 2010: old aged persons (65 years and above), during the same period, recorded a considerably higher increase from 254,937 to 678,731 (Fig. 1). While the absolute figures are astonishingly increasing, percentage of old aged population rose slowly as compared to any other population in transition. Such an ageing process took place along technological advances, improvement in infrastructure and migration flows. All these changes influence age structure, sex composition, and, thereby, ageing population in many ways including life style. Although population of the country could be distinguished into native and foreign in terms of size and characteristics, comparisons are impractical and misleading. It is because foreigners in the country are on labor contract, a majority are without family provisions, thus having a skewed age distribution^2,4,24,35,36^.

**Figure 2:**
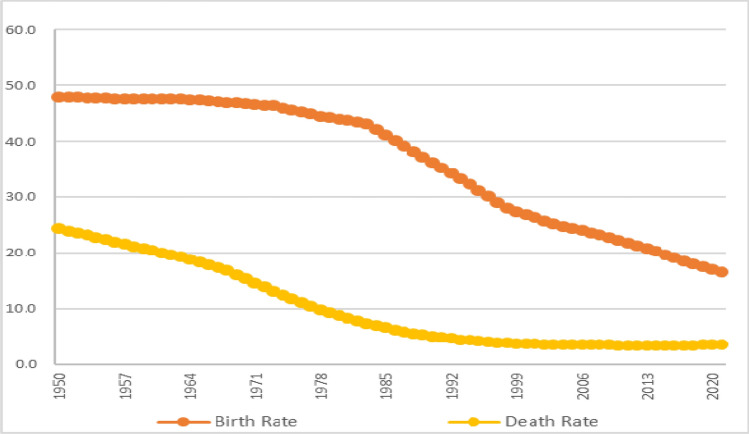
Demographic Transition in Saudi Arabia. Source: Saudi Arabia—Historical Birth Rate Data (https://www.macrotrends.net/countries/SAU/saudi-arabia/birth-rate) (). Note: Caution is required as these figures are based on total population (natives and expatriates); most of the births and deaths of expatriates are recorded in their respective countries, and not in Saudi Arabia. But the denominator (mid-year population) might be inclusive of both native and expatriate population.

The transition that the country has gone through, despite demographic lag and resistance to fertility decline (pronatalist policy), characterizes a sharp decline in mortality from 1950 to 1978 (crude death rate of 24.4–9.8); declined further thereafter till 2000 (3.8); and static low since then (3.4/3.5). Birth rate in the country varied: remained high since 1950 (48.0); static till 1970 (46.7); slowly declined till 1984 (42.4); sharp decline, thereafter, till 2000 (26.9); and a fast decline till 2021 (16.6). As seen in Fig. 2, there was a wide gap even in the pre-transitional stage (1950–1970); widened during the early transitional stage (1970–1985) and declined thereafter in the mid transitional stage (1985–2000) and in the late transitional stage (2000–2021). Early transitional stage, as depicted, illustrate birth rate falling at a low pace but death rate falling remarkably. Declines in birth rate continued, thereafter, in the next stage too, but that of death rate was lesser, due mainly to the already achieved low rates. The next stage which could be divided into two: first (2000–2010) and second (2010–2021): the former characterizes a fast decline of birth rate whereas the latter has a stalled decline, death rate remained low at both these phases. This, probably, is a landmark in transition, where the birth rate decline was slow but steady. Such a late transitional stage continues for a period till reaching a replacement level fertility, have direct and indirect implications for older persons and their families^14,37,38^. Similar transitions leading to ageing process were illustrated earlier^16,26,39^.

### Demographic transition as the base

Demographic transition, explained above, lead to population growth that changes population structure, distribution and composition. Gap between birth and death elaborate the volume of natural increase; high in pretransitional stage, higher during early transition, lower during mid transition, and thereafter narrowed during late transition. Along with the transition is the change in age structure showing a shift from a baby boom to youth/adult bulge (demographic dividend), which further proceed to retirement boom, and thereafter, to old age boom: a process that Saudi Arabian population currently passes through^25^. On structural ground, while plotting age pyramid, this process reflects in a constriction, narrow base but bulged center. This type of age pyramids, termed as constrictive pyramids, characteristic of transition period, look different from expansive pyramids having broad base that narrows upwards. Later, at the end of the transition, age pyramids turn to be cylindrical, as that of Western countries. Such changes are reflections of social and economic developments and transitions, hence leading to transformations in quality of life.

Due to the transition on demographic front along with improvements in socio-economic and service infrastructure, there are improvements in survival or life expectancy, which impact upon age structure—children, youths, adults and elderly (the broad age groups). This effect on their numbers directly, influencing life style and behavior, probably, lead to changes in infrastructure—education, health, employment, transport, and other service utilities. Saudi Arabian population as a whole has an age structure, which cannot be theoretically explained for to its shape which was caused mainly due to the expatriate population brought to fill the demand–supply gap in labor force. Each stage in demographic transition demonstrate socio-economic and cultural hanges beginning with an agrarian (pretransitional stage) transforming into an ultramodern (post transitional stage) life style and quality of life. Hence, demographic transition is simply an explanation of improvement in infrastructure and development leading to progress towards quality of life dimensions, stage by stage. Structural concerns of similar type exist in other parts of the world, even in the Arab region^6,26,40^. The real picture shall be brought out while distinguishing native from foreign population. This is caused due to the working age population brought from other countries, that too, without family provisions. Thus, the native population age structure receives special attention while explaining demography.

### Changes in population pyramid

**Figure 3:**
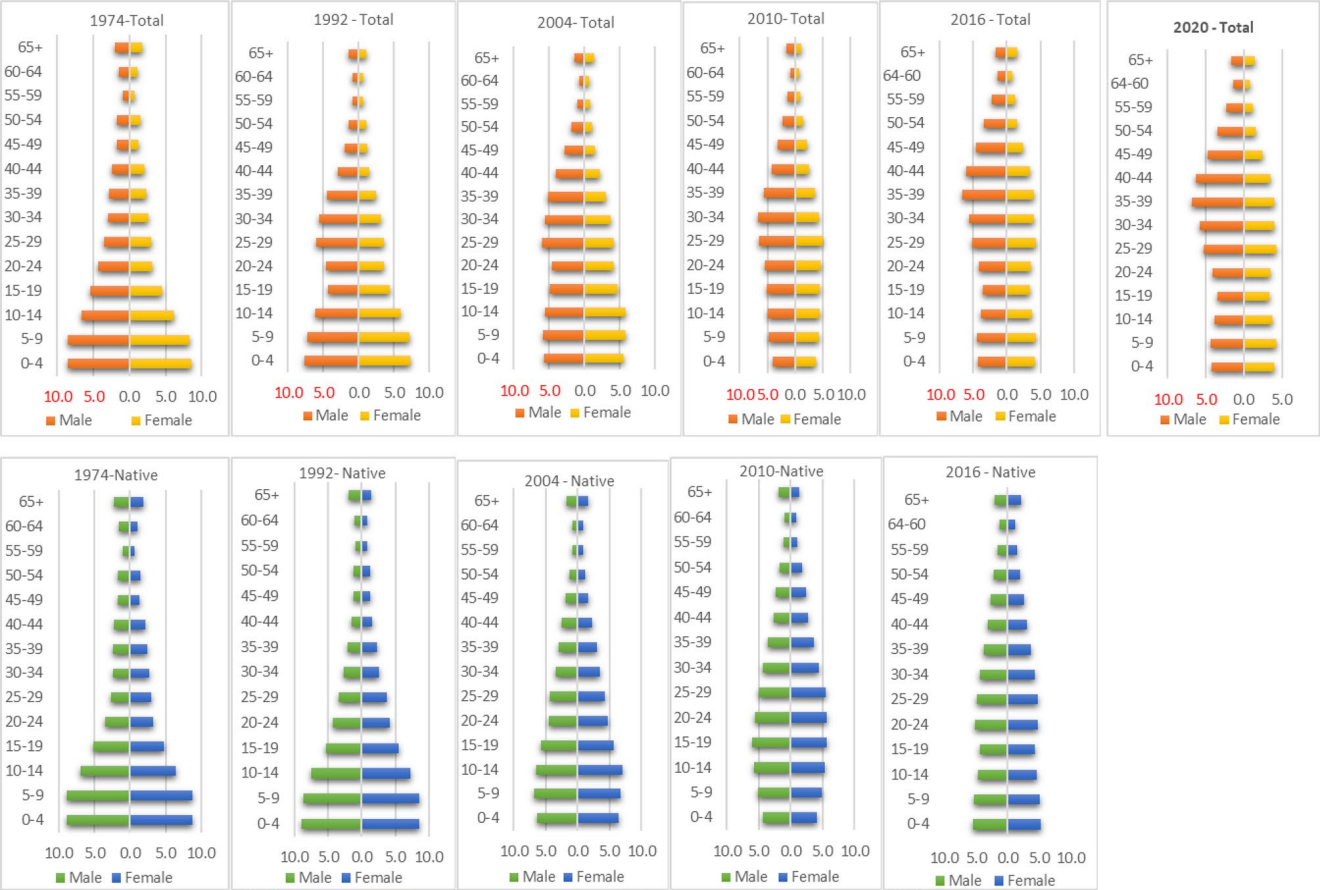
Age structural changes in the total population and the native population, over the years.

While the overall population age structure is leaned towards males of working ages, that of the native population is constrictive (Fig. 3). In short, population of the country cannot be considered together due to their residential—citizenship—status influencing duration of stay coupled with feeling of long term safety and security. Majority of the immigrants living in the country invest their income in their native land for future (after return) to settle and live in old age or superannuation.

Although, the total population could be explained as due to the immigrant load, dominated by adult males, without their family members, the native population characteristics showing a rapid change expand the age group of 50–64 years, shall soon impinge upon the next category—old age. As points out, this window of opportunity stagnates after 15 years^27^. Thus, an optimum use for favorable economic growth potential focusing on savings and investments are necessary through policies to facilitate a sustained future of quality technical education and development.

Native population, born, brought up and continue living with their families shows a theoretical demographic pattern where the nature of change of population structure through transition in births and deaths lead to a future of population ageing, although, the old aged did not change in terms of percentages—neither males nor females^2,5,41,42^. Such indications of population ageing at a faster rate, in the near future, call for serious thoughts and sustained social policies and plans that goes beyond welfare measures and medical services, to ensure age family integrated approach, promoting a society for all ages, welfare society, needs of ageing, etc.,^1,13,14,28,30–34,41,43,44^.

### Percentage distribution to infer ageing trends

Changes were observed during the periods of 1974, 1992, 2004, 2010, 2016 and 2020 that the percent of children (0–9 years) declined as 35.4, 34.6, 26.4, 18.5, 21.0, and 16.9 respectively; adolescent/youth (10–24 years) slightly increased and then decreased as 30.1, 33.9, 34.3, 34.0, 28.0, and 22.1; young adults (25–49 years) rapidly increased from 23.0, 22.3, 29.9, 36.6, 37.1, and 47.1; and late adults (50–64 years) showed minor changes in from 7.5, 5.9, 5.9, 7.6, 9.7, and 10.8 (Table 1). Such a delayed structural transition might have been due the scenario of the previous decade combined with cultural concerns and social demands: causes of immigration^2,45^. But, in case of native population, changes are of a different nature: rapid decline in children and adolescent-youth but remarkable increase in young adults and later adults with a no change in old aged population. This is crucial in demographics that a decline in childhood and adolescent/youth influenced to raise adult population but not yet accelerated to old aged. This could, probably, take more time, waiting time, to ageing.

**Table 1:** Percentages of population by broad age groups, total and native.

	Native					Total				
	0–9	10–24	25–49	50–64	65 +	0–9	10–24	25–49	50–64	65 +
**1974**										
Male	32.1	30.7	25.4	7.8	3.9	34.6	30.4	22.4	8.2	4.3
Female	36.0	29.5	24.2	6.7	3.7	36.2	29.7	23.6	6.7	3.8
Total	34.0	30.1	24.8	7.3	3.8	35.4	30.1	23.0	7.5	4.1
**1992**										
Male	26.8	27.1	37.8	5.7	2.6	34.7	33.8	21.6	6.3	3.7
Female	33.2	31.9	27.3	5.0	2.5	34.6	34.0	23.1	5.5	2.9
Total	29.6	29.2	33.2	5.4	2.6	34.6	33.9	22.3	5.9	3.3
**2004**										
Male	20.9	27.2	42.8	6.5	2.6	26.5	33.7	30.3	6.0	3.6
Female	25.5	33.1	32.8	5.6	3.0	26.3	35.0	29.4	5.9	3.4
Total	22.9	29.8	38.3	6.1	2.8	26.4	34.3	29.9	5.9	3.5
**2010**										
Male	15.5	27.6	46.3	8.1	2.6	18.8	34.3	35.4	7.9	3.6
Female	18.7	31.6	40.3	7.0	2.4	18.2	33.8	38.0	7.4	2.7
Total	16.9	29.3	43.7	7.6	2.5	18.5	34.0	36.6	7.6	3.2
**2016**										
Male	15.2	20.2	49.3	12.3	3.0	21.0	28.2	36.9	9.9	4.0
Female	19.7	25.4	42.8	8.5	3.6	21.1	27.8	37.2	9.6	4.4
Total	17.1	22.4	46.5	10.7	3.2	21.0	28.0	37.1	9.7	4.2
**2020**										
Male						14.9	19.8	49.9	12.4	2.9
Female						19.6	25.2	43.1	8.5	3.6
Total						16.9	22.1	47.1	10.8	3.2

Ageing issues are not cropped up in the country due to values of tradition, practice, and community bonding facilitating family solidarity and filial responsibility. However, there are indications of a rapid growth of old aged in the population soon or later from its low percentage, as evidenced from the constriction of population pyramid, from early 2000s. There is an increase in the native population share of early adults and late adults with a decline in childhood and adolescent-youth age. At the same time, the change in old aged is not visible, hopefully, requiring a further period (a decade).

### Indices of ageing in explaining ageing process

Ageing in a population could, positively, be explained through indicators such as age dependency ratio, index of ageing and median age, as explained^2^. An increase on these indicators shows ageing process in a population (Table 2). Although it is a slow movement, currently, it is expected to raise faster in this decade. Vividly, these rates have changed from 8.6 to 6.4 in age dependency; 15.7 to 25.5 in median age; and 8.3 to 13.8 in index of ageing, between 1974 and 2020 and 144.5 to 169.9 in ageing of the aged between 1992 and 2016, among natives. There are minor differences between males and females in these rates.

**Table 2:** Age related indices illustrating ageing of population.

	Age dependency ratio	Median age	Index of ageing	Young-old to old-old	Median age (65 +)
**1974**					
**Native**					
Male	9.1	16.0	9.0		
Female	8.0	15.3	7.6		
Total	8.6	15.7	8.3		
**Total**					
Male	7.6	17.6	8.8		
Female	7.7	15.5	7.5		
Total	7.7	16.7	8.1		
**1992**					
**Native**					
Male	7.8	15.3	7.4	148.3	73.3
Female	5.9	15.4	5.8	139.8	73.6
Total	6.9	15.4	6.6	144.5	73.4
**Total**					
Male	4.4	22.6	6.9	153.7	73.1
Female	5.0	16.6	5.4	142.0	73.6
Total	.6	19.7	6.1	148.5	73.3
**2004**					
**Native**					
Male	6.3	19.6	9.0	156.1	72.9
Female	6.0	19.2	8.4	199.2	72.3
Total	6.2	19.4	8.7	175.3	72.6
**Total**					
Male	4.0	25.9	8.5	166.1	72.6
Female	5.1	20.4	7.7	199.0	72.3
Total	4.4	23.4	8.1	180.8	72.4
**2010**					
**Native**					
Male	5.5	23.6	12.2	228.3	70.7
Female	4.0	24.1	9.3	212.6	71.5
Total	4.7	23.9	10.8	221.6	71.1
**Total**					
Male	3.5	28.0	10.6	238.4	70.6
Female	3.5	24.8	8.1	215.8	71.5
Total	3.5	26.7	9.4	228.8	71.0
**2016**					
**Native**					
Male	6.1	25.4	13.2	167.2	72.6
Female	6.7	25.6	14.3	172.5	72.3
Total	6.4	25.5	13.8	169.9	72.4
**Total**					
Male	4.0	32.8	13.5	199.5	71.8
Female	5.3	27.4	12.6	189.1	71.9
Total	4.5	30.5	13.1	194.5	71.9
**2020**					
**Total**					
Male	3.9	33.1	13.6	199.1	71.8
Female	5.3	27.6	12.6	183.1	72.2
Total	4.4	30.8	13.1	191.3	72.0

Age dependency ratio, calculated as number of aged 65 years and above to 100 persons of working age declined drastically during 1974–2020, which indicates a widening of the middle of the age pyramid, which is fast approaching to the top^25^. A similar observation could be made from the median age too that it is on a rapid increase, especially of the native population. Male–female difference is unnoticed except a slightly higher gain in females. Even the total population, as a whole, have also gained in this parameter. That is, expatriates are older than natives in the country, which could also be due to the lesser number of children.

Index of ageing calculated as older persons to 100 children of age below 15 years, increased rapidly explaining that the proportion of old aged, comparatively, increasing in the population. It shows the fertility trends as a determinant of population ageing. This trend is common to other Arab countries also, and which is expected to increase further with advancement in demography^46^.

### Other supportive data on ageing and health

National elderly survey, 2017 and national household health survey 2018 give feedback on old age issues in the country for both native and total population. These data have been interpreted in this context to explain compression of morbidity, medical support and assistance, and mortality (Table 3). It could be realized that morbidity compressed to progressive ages that an increasing percentage suffering from various ailments accounting for comorbidity are in the old age. Compared to young old categories, old-old category is highly morbid, higher in 80 + age group. Both diabetic and hypertensive increased from 65–69 to 75–79 years, its percentage declines after that, in 80 + age group, both in males and females.

**Table 3:** Inferences from surveys.

Age group	65–69	70–74	75–79	80 +	Total
National elderly survey—2017					
**a. Compression of morbidity in old ages—Native population**					
**Total**					
Heart disease	7.43	10.22	10.88	15.71	10.46
Diabetic	35.04	40.75	44.47	38.92	38.93
Hypertension	33.98	36.87	45.44	43.36	38.61
Cholesterol	9.95	9.96	11.02	12.3	10.62
Depression	0.45	1.13	0.39	1.73	0.89
Arthritis	15.51	16.11	22.29	25.32	18.84
Asthma/chronic pneumonia	3.57	4.26	6.99	9.18	5.49
Fractures	0.73	0.66	0.77	1.32	0.84
Alzheimer’s disease	0.13	1.26	1.76	4.29	1.56
Gastroenterology	2.55	4.18	4.83	5.61	3.99
Kidney diseases	1.97	1.69	2.33	2.87	2.15
Other chronic diseases	2.38	3.09	3.64	3.96	3.11
Total	113.71	130.19	154.83	164.57	135.48
**Male**					
Heart disease	7.87	10.20	11.75	14.00	10.40
Diabetic	37.12	36.40	43.36	36.47	37.86
Hypertension	31.18	29.74	39.71	37.74	33.60
cholesterol	9.48	5.79	8.42	10.48	8.53
Depression	0.53	0.21	0.47	0.13	0.35
Arthritis	13.26	12.14	16.87	20.30	15.02
Asthma/chronic pneumonia	3.47	2.51	5.25	6.03	4.04
Fractures	0.83	0.72	0.47	0.83	0.74
Alzheimer’s disease	0.14	0.41	2.42	3.92	1.37
Gastroenterology	3.25	2.66	1.33	4.39	3.00
Kidney diseases	2.41	2.64	2.36	1.51	2.28
Other chronic diseases	2.34	2.13	2.24	3.91	2.59
Total	111.87	105.57	134.66	139.71	119.79
**Female**					
Heart disease	7.02	10.24	10.03	17.30	10.51
Diabetic	33.08	44.99	45.56	41.21	39.94
Hypertension	36.62	43.83	51.03	48.59	43.40
cholesterol	10.39	14.03	13.55	14.00	12.61
Depression	0.38	2.04	0.31	3.24	1.39
Arthritis	17.65	19.98	27.58	30.01	22.50
Asthma/chronic pneumonia	3.66	5.96	8.68	12.13	6.87
Fractures	0.64	0.61	1.07	1.78	0.94
Alzheimer’s disease	0.13	2.08	1.12	4.63	1.74
Gastroenterology	1.89	5.66	8.26	6.74	4.94
Kidney diseases	1.56	0.77	2.30	4.13	2.02
Other chronic diseases	2.42	4.02	5.00	4.01	3.60
Total	115.44	154.21	174.50	187.77	150.47
**b. Older persons needing support**					
**Total**					
Take medicine	6.70	8.38	7.33	17.10	39.51
Personal care	7.23	8.21	7.57	26.04	49.06
Mobility and movement	9.28	10.64	9.26	20.32	49.50
Eat and drink	3.31	3.96	3.69	11.19	22.15
Total	26.52	31.20	27.85	74.67	160.23
**Male**					
Take medicine	3.84	4.37	4.94	13.86	27.00
Personal care	3.60	6.08	7.00	19.70	36.37
Mobility and movement	6.20	7.34	6.04	15.58	35.17
Eat and drink	1.96	2.39	2.77	8.03	15.15
Total	15.60	20.18	20.74	57.16	113.68
**Female**					
Take medicine	9.41	12.17	9.59	20.17	51.34
Personal care	10.67	10.22	8.12	32.04	61.06
Mobility and movement	12.19	13.76	12.30	24.80	63.04
Eat and drink	4.58	5.45	4.56	14.19	28.78
Total	36.84	41.60	34.56	91.21	204.21

	Diagnose				
	Cardiovascular disease	Cancer	Diabetes	Hypertension	
National household health survey—2018					
**a. Morbidity**					
**Native population**					
Percentage of 65+ age group out of the total number of affected persons	40.36	21.88	29.07	33.52	
Percentage of total 65+ population	11.40	1.00	50.65	53.26	
**Total population**					
Percentage of 65+ age group out of the total number of affected persons	35.15	19.97	23.02	27.45	
Percentage of total 65+ population	10.46	0.88	48.30	51.08	

	Males	Females	Total		
**b. Mortality**					
65+ years	19,139	17,745	36,884		
Total deaths (all ages)	49,137	31,109	80,246		
Percentage of total deaths	38.95	57.04	45.96		
Percentage of total 65+ population	1.86	1.73	3.59		

On the other hand, other morbid conditions increases in percentages with progressing age, more clearly in females. Arthritis, heart diseases, Asthma/pneumonia, gastrointestinal diseases, Alzheimer’s disease, and kidney diseases are common ailments apart from physical conditions such as diabetes, hypertension, and cholesterol. Moreover, all these diseases have its higher frequency and thus percentages in females making them as more vulnerable and at risk in old age, as pointed out—the most popular feminine nature of ageing^46,47^. Old aged suffering co-morbidity are explained^48^, where the resource intensive primary health centers could be equipped to cater to those patients.

As per the household health survey that assessed major morbid conditions such as cardiovascular disease, cancer, diabetes, and hypertension has 40.36%, 21.88%, 29.07%, and 33.52%, respectively, as older persons share, exclusively for native population. While this share of affected persons within the old aged population calculated using 2016 population totals, they are 11.4%, 1.00%, 50.65%, and 53.26%, respectively. These statistics show the morbidity load in old age, considered to be the compression of morbidity, to the later years of life. In case of the total population, these percentages are lesser, which could be because of migrant behaviors during ill-health, and related uncertainty. Survey results on morbidity also explains these instances during senile ages, which comes out to be very high. However, older persons mortality remains as 3.59 percent of total population of the age but 45.96 of total deaths. Hence, a substantial number of older persons with morbidity and disability demand care, concern, and support not only from kith and kin but also from wider society, as has been emphasized^29,49–51^. Both supports and co-residences decline in Saudi Arabia along socioeconomic changes impacting self-esteem, as evidenced^17,52^. Apparently, self-perceived health is influenced through programs and leisure time activities promoting active and healthy ageing^15,53^.

### Proposed strategies

There are a variety of strategies adopted in population ageing with a view to address demands (such as skill development, university of third age, domestic support services, respite care facilities and senior citizen clubs) in addition to service, education, research, and sociocultural supports, where an upgraded, coordinated, primary health care system could cater to health needs and demands^3,34,37^. Hence, health and nutrition, housing and environment, family, social welfare, and income security and employment, as explained in plan of actions by the United Nations^30–32^, seeks importance in the cultural context in ensuring quality of life of older persons and enabling them to live actively and independently in communities. Provisions of adequate health care and social security by upholding social support systems –informal and formal–enhancing abilities of relatives to take care of older persons within the family are ways forward to meet the challenges of population ageing^46^. Further, with welfare systems not only the older persons but also the families with an older person shall be facilitated to lead a quality life, through concerted population and public health policies and programs as pointed out^2,13,14^. Such welfare strategies accounting the contributions of older persons in the society, giving importance to a person centered approach shall facilitate achievement of better levels of health in ageing characterized by reduction of disabilities, chronic diseases, and premature mortality; instigating increased responsiveness to risk factors; investing more on human and financial resources; and upholding national capacity for geriatrics^22,44,54^.

Moreover, with elderly friendly infrastructure and services, quality of life of older persons could be enhanced by making them self-reliant and less dependent, thereby ease care givers through institutions and networks of services, conforming with local culture and norms enhanced by health infrastructure—well-trained medical and paramedical personnel^28,34^. In addition, self-reliance of older persons necessary for their social participation to be ensured through strengthening informal care systems that enable them to lead a self-determined, healthy, and productive life helpful for utilizing skills and abilities, which would require pouring welfare measures to the carers and their families^26,33^.

Available demographic bonus, of today, to be maximum utilized and invested for the turbulence period that follow with a faster ageing resulting in a stagnated working age^27^. Co-morbidity, leading to compression of morbidity, is to be handled at resource intensive primary health care services^48^. This is a strategy along with sustained healthy life style campaigns having long term benefits in ageing societies in ensuring a quality of life^38^, which could be adopted in the country. Feminine nature of ageing might be encountered by creating female friendly employment, particularly in hospitality and service sectors so as to increase their work participation rate^55^. Additionally, bridge jobs might mitigate the negative shocks of retirement satisfaction while combined with various other models of retirement frameworks^56^. Thus, a theoretical approach of building structures and systems to accommodate and sustain community living with the kith and kin of older persons to be prioritized and promoted in the country, in a futuristic manner.

## Conclusions

Saudi Arabia is ageing slow but steady: patterns are observed only among the native population due to the restrictions applicable to foreign labor force in demographics, family, and dependent status. A close look at the native age distribution and age pyramid reveals changes as theorized in the demographic transition. A wide gap between birth and death characteristic of early transition period changes the age pyramid and further constrictions in the mid transition stage, proceeding to the current late transition where pyramid constricted further. This trend continues for a period, thereby, the age pyramid taking a cylindrical shape, hopefully, in the coming 20 years.

Percentage distribution of broad age groups, shrinking of child and adolescent-youth age with a simultaneous expansion of working age, especially of 20–49 years show the trend of demographic dividend. This window of economic opportunity remains there for a short period of another 15 years, moving slowly to higher levels of age structure, that is to retired old age of 65 years and above. Moreover, this trend is confirmed through indices such as age dependency ratio, median age, and index of ageing. Still, the ageing of the aged population or median age of aged do not started showing a trend, due mainly to the dividend, which might be reversed soon, in the decade.

Still, with the available statistics, it could be inferred about compression of chronic morbidity; longer years of retired old age life; lifestyle diseases comorbidity; increased demand for care; and support outside of the informal care system are challenges to add life to years. As seen elsewhere, here too, the feminine nature of ageing make older women as more vulnerable.

In this context, major programmatic experimentations, all over the world, in line with United Nations plan of actions are advised to be considered in the country with special regards to the culture, traditions, values and social order. Without hindering any of the inbuilt structures and systems, the old aged should be cared by maintaining their dignity and independence so as to build a society for all ages fostering an age family integrated approach. It should focus on informal care systems, for which care, support, and guidance are needed to family, in addition to welfare schemes for caring older persons. Additionally, a system of informal care can be built, such as home care facilities, home nursing, domestic support, respite, senior citizen clubs, neighborhood support schemes, and mobile clinics and nutrition advices (Supplementary information S1).

## Supplementary Information

Supplementary Information.

## Data Availability

All data generated or analysed during this study are included in this published article and its supplementary information files.
